# Effect of row spacings on soil nematode communities and ecosystem multifunctionality at an aggregate scale

**DOI:** 10.1038/s41598-020-61498-x

**Published:** 2020-03-16

**Authors:** Guizong Zhang, Xinchang Kou, Xiaoke Zhang, Wei Bai, Wenju Liang

**Affiliations:** 1 Institute of Applied Ecology, Chinese Academy of Sciences, Shenyang, 110016 China; 2 Tillage and Cultivation Research Institute, Liaoning Academy of Agricultural Sciences, Shenyang, 110161 China; 3 School of Geographical Sciences, Northeast Normal University, Changchun, 130024 China; 4 University of Chinese Academy of Sciences, Beijing, 100049 China

**Keywords:** Food webs, Agroecology

## Abstract

Effect of crop row spacing on the belowground ecosystem, especially at an aggregate scale, remains unexplored. To explore how row spacing influenced nematode community and ecosystem function at the aggregate scale, four row spacings i.e. equidistant-row (ER, 50 cm-inter-row distance, 33 cm-intra-row between plants in each row) and non-equidistant-row including NR1 (100 cm + 50 cm row distance and 22 cm intra-row), NR2 (100 cm + 50 cm inter-row and 25 cm intra-row), and NR3 (60 cm + 40 cm inter-row and 33 cm intra-row) were compared, and four soil aggregate fractions i.e. >2 mm, 1–2 mm, 0.25–1 mm and <0.25 mm were separated. Row spacing did not impact C and N, but significantly influenced P. The regulation effect of acid phosphatase on soil available P was aggregate-scale dependent. Nematode faunal analysis indicated that NR3 within 0.25–1 mm was less disturbed or relatively undisturbed environments. Structural equation model showed row spacing pattern directly affected multifunctionality, while aggregate fractions indirectly contributed to multifunctionality mainly by regulating the richness of total nematodes and trophic groups. It was concluded that NR3 had potential to construct more stable food web, and therefore was possibly the suitable planting pattern.

## Introduction

Although agronomic management options are diverse, such as no tillage, rotation tillage, their aims are all unanimous, to increase yield by regulating all positive abiotic and biotic factors to meet crop demands^1^. Among these management options, manipulating row configuration (inter- and intra-row spacing, i.e. the distance between rows, and the distance between the plants in a row, respectively) to alter crop spatial arrangement is feasible, whose objective is to get more solar radiation and alleviate soil nutrient competition among crops etc^2^. However, the controversy over the optimum row spacing has been ongoing for many decades because the evaluation on row spacing depend upon local growth environment and management factors^3^. From an ecological perspective, rational row spacing is expected to increase radiation interception (RI, intercepted by the canopy, directly linked to net photosynthetic production) and to maximize ecosystem function because available light, water and nutrients are optimally allocated^4^. Compared with equidistant-row (ER), non-equidistant-row (NR) arrangement is characterized by higher leaf area index and radiation use efficiency^5^. The NR has been proposed as an alternative practice in order to alleviate crop crowding stress, decrease plant-to-plant competition and increase light penetration to lower plant leaves^6^. NR can also improve nitrogen use efficiency via increasing plant nitrogen uptake^7^. Furthermore, variation in row spacing would alter the spatiotemporal distributions of root length density and root mass, and therefore affect soil pore and aggregate formation which are all important for crops to acquire soil nutrients^7–11^. As mentioned above, many studies on the effect of row spacing on crop, light radiation use efficiency and soil nutrient and structure are well conducted, yet the effect of row spacing on soil biota communities remains unclear.

Ecosystem functions such as soil organic matter decomposition and nutrient cycling, are mainly dependent on soil biota, which are critical for crop nutrient uptake in agroecosystems^12^. Microbes as the important soil biota secrete enzymes in the soil thus, to some extent, soil enzymes are good surrogates of microbial activity and have been used as indicators of microbial nutrient demand^13^. As a critical fauna of the soil food web, nematode affects ecosystem functions by grazing certain microbes and then altering microbial community composition and distribution, and ultimately changing soil nutrient turnover such as carbon, nitrogen and phosphorus mineralization^14^. Thus, the nematode community composition and diversity can either positively or negatively affect ecosystem functions. Additionally, spatial scale is one of the important factors for studying soil ecosystem functioning. Besides row spacing as small spatial scale, soil aggregates as micro-scale also affect soil biota community^15^. It is critical to explore the soil biotic community within soil aggregates in order to gain a better understanding of their roles in ecosystem functioning^16^. For example, geometrical characteristics within soil aggregates, such as soil pore volume, shape, connectivity and tortuosity of pathways all have an effect on nematode community composition through affecting their acquisition of microbial food sources^17^. The relatively small pore size of microaggregates (<0.25 mm) makes them inaccessible to larger-sized nematodes^18^, therefore protects microbes living inside from their predators.

However, less is known about the effect on soil biota, especially nematodes and the corresponding multifunctionality at small spatial scale (row spacing) and micro-scale within aggregates. Therefore, we analyze the effects of row spacing on soil nematode community composition and ecosystem multifunctionality at an aggregate scale in a field experiment to determine the relationship between nematode community composition and ecosystem multifunctionality within soil aggregates. Based on above, we hypothesized that compared with equidistant-row (ER), non-equidistant-row (NR) may improve soil ecosystem multifunctionality, and soil nematode communities vary with different spatial scales (row spaces and aggregates), and their variation will correlate with multifunctionality based on soil indicators.

## Results

### Soil pH and proportion of soil aggregate fractions

**Figure 1:**
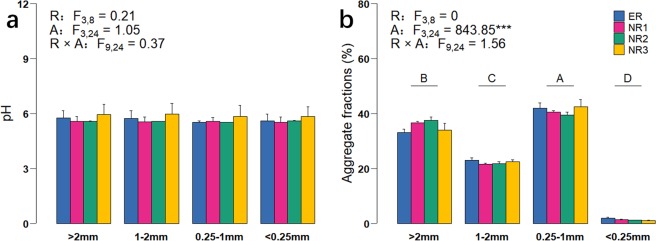
Effects of row spacing on soil pH (**a**) and the proportion of different aggregate fractions (**b**). Error bars indicate standard errors. F and P values from a two-way ANOVA on the effects of row spacing (R) and aggregate fraction (A) and their interactions (R × A) are also presented, significance levels are as follows: *P < 0.05, **P < 0.01, ***P < 0.001. Different uppercase letters on the horizontal line indicate significant differences among aggregate fractions regardless of row spacing at P < 0.05.

Neither row spacing nor aggregate fraction impacted soil pH (Fig. 1a). Only aggregate fraction but not row spacing effect was significant on the proportion of aggregate fractions (Fig. 1b, P < 0.01). Under all row spacings, the rank of the aggregate fraction proportions from high to low was 0.25–1 mm, >2 mm, 1–2 mm and <0.25 mm.

### Multifunctionality based on soil indicators

**Figure 2:**
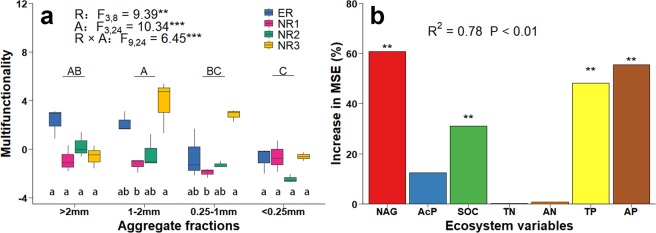
Effects of row spacing on multifunctionality (**a**) and main contributors of multifunctionality showed by the Random Forest mean contributor importance (% of increase of MSE). (**b**) Error bars indicate standard errors. F and P values from a two-way ANOVA on the effects of row spacing (R) and aggregate fraction (A) and their interactions (R × A) are also presented, significance levels are as follows: *P < 0.05, **P < 0.01, ***P < 0.001. Different uppercase letters on the horizontal line indicate significant differences among aggregate fractions regardless of row spacing at P < 0.05.

The row spacing, aggregate fraction and their interaction significantly impacted multifunctionality (Fig. 2a, P < 0.01). NR3 had higher value of multifunctionality than NR1 in both 1–2 mm and 0.25–1 mm (P < 0.05). Among different soil aggregate fractions, the value of multifunctionality was lower in <0.25 mm than>2 mm and 1–2 mm (P < 0.001). Random forest analysis showed that β-N-acetylglucosaminidase (NAG, 61%), available phosphorus (AP, 55%), total phosphorus (TP, 48%) and soil organic carbon (SOC, 31%) are the main contributors of multifunctionality (Fig. 2b, R^2^ = 0.78, P < 0.01). For the indicators of ecosystem functions, aggregate fraction effect on NAG and SOC were also significant (P < 0.05), with the lowest value of NAG in >2 mm and SOC in <0.25 mm aggregate fractions (Fig. S1a,c). Both row spacing and aggregate fraction had significant influence on TP and AP (P < 0.05), but not on TN (Fig. S1d,f,g). Compared to ER, the amount of TP was higher under NR3 within 1–2 mm and 0.25–1 mm aggregate fractions (P < 0.01). Different from TP, there is opposite trend in AP with lower values under NR3 within the same aggregate fractions (P < 0.001). Acid phosphatase (AcP) under NR3 were all lower in >2 mm, 1–2 mm and 0.25–1 mm than those under ER (Fig. S1b, P < 0.001).

### Nematode community composition

**Figure 3:**
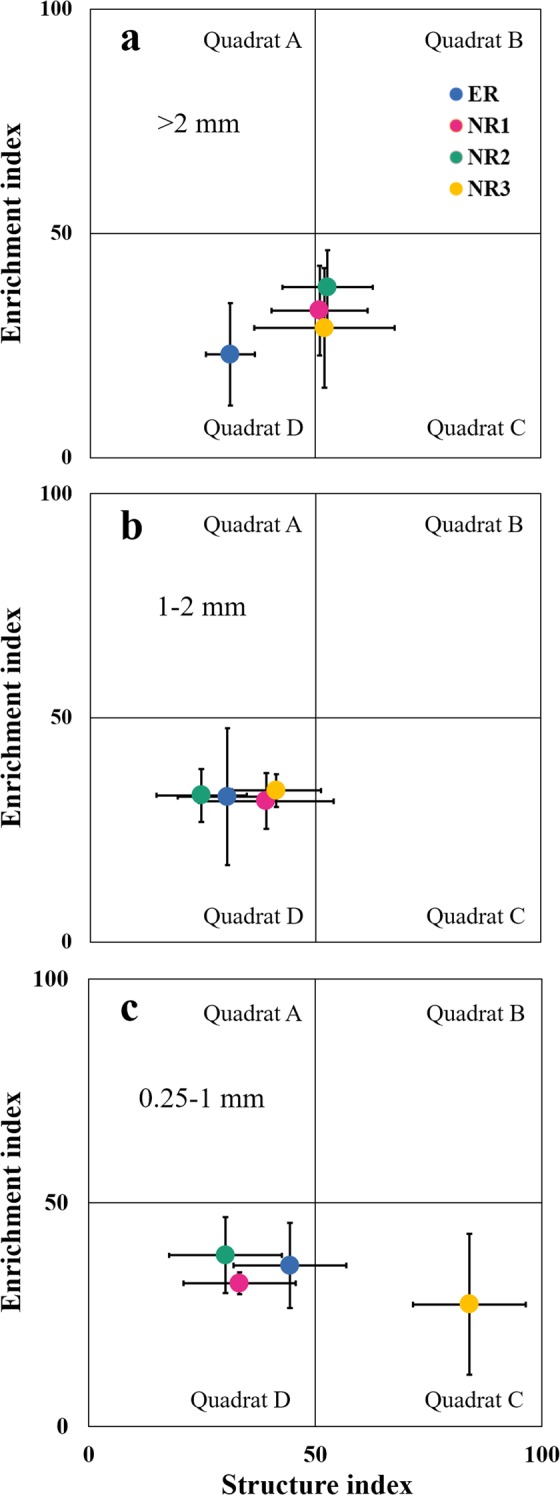
Nematode faunal analysis in >2 mm (**a**), 1–2 mm (**b**) and 0.25–1 mm (**c**) aggregate fractions under different row spacing treatments. Mean and standard error for enrichment and structure indices are shown. Faunal analysis for <0.25 mm was not shown because value of SI and EI of each row spacing is zero.

Significant aggregate fraction effect was observed in the richness and abundance of total nematode community and the four trophic groups (P < 0.01). Both richness and abundance of total nematode community, bacterivores (Ba), plant-parasites (PP), and the richness of fungivores (Fu) were lower in 0.25–1 mm and <0.25 mm compared to 1–2 mm aggregate fractions (P < 0.01) regardless of row spacing (Fig. S2a–e,g,h). Only 1 genus belonged to Ba within <0.25 mm aggregate fraction was found under ER and NR2 (Fig. S2c). The nematode guilds with cp value 4–5 mainly composed of *Aporcelaimellus* (from omnivore-predators, OP), *Discolaimus* (OP), *Eudorylaimus* (OP), *Longidorus* (PP) and *Pungentus* (OP) was the highest in 0.25–1 mm aggregate fraction under NR3 (Fig. S3). Soil nematode faunal analysis showed that most plots of ER, NR1 and NR2 in 0.25–1 mm aggregate fraction were situated in quadrat D, which suggested the severely disturbed environments, whereas those of NR3 in quadrat C indicated less disturbed or relatively undisturbed environments (Fig. 3).

### Structural equation model

**Figure 4:**
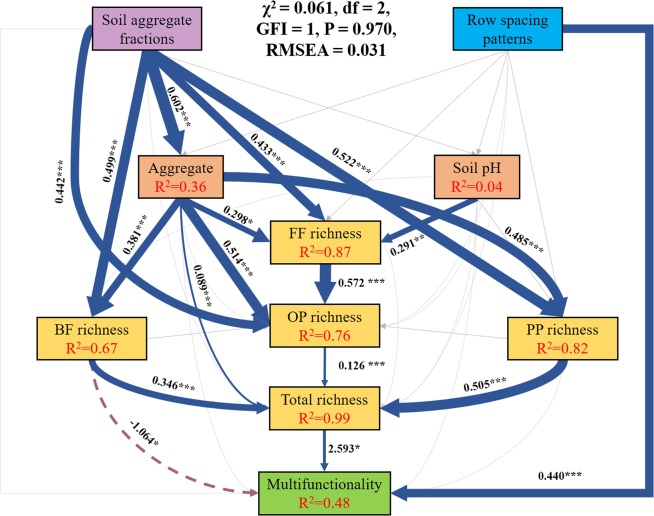
Structural equation model linking row spacings and soil aggregate fractions to nematode community characteristics. Solid line indicates positive effect, dotted line indicates negative effect. BF: bacterivore; FF: fungivores; PP: plant-parasites; OP: omnivore-predators.

Structural equation model (χ^2^ = 0.061, df = 2, GFI = 1, P = 0.970, RMSEA = 0.031) showed that row spacing patterns had direct effect on multifunctionality (P < 0.01), while soil aggregate effect indirectly correlated with multifunctionality through modifying the proportion occupied by different aggregate fractions, controlling trophic group richness, and then total richness (Fig. 4).

## Discussion

In our study, the effect of row spacing on both soil aggregates and SOC was not observed. Soil aggregate stability was positively correlated with SOC that are major binding agent especially for aggregate fraction with 0.25–2 mm^19,20^. However, the main differences caused by row spacing changes are the plant spatial distribution and then the competition among plants for light and nutrients^2^. Carbon synthesized by photosynthesis has more direct effect on aboveground plant and indirect impact on soil ecosystems^21^. The accumulation in soil carbon is a relative long-term process and does not respond to row spacing variation in the 3-year field experiment. These reasons may result in unobvious variation in soil aggregates and soil organic carbon among different row spacing treatments.

In contrast to C, most P in soils are presented in insoluble forms, and phosphate are the main inorganic form of P, which is available to plant uptake. Acid phosphatase (AcP) was primarily secreted by soil microbe and plant roots, and has the regulation effect on soil organic P hydrolysis to release phosphate^22^. Both row spacing and aggregate fraction significantly impacted AcP and soil P. Compared to ER, the AcP activity in NR3 was significantly lower in >0.25 mm. There was a strong negative relationship between AcP activity and pH (P < 0.01), and a slight increase in pH (from 5.6 in ER to 5.9 in NR3 in >0.25 mm) may be enough to inhibit AcP activity in our study. Significantly higher AP content and lower AP activity were found in NR3 than in ER at >2 mm. The opposite trend between AP and AcP was consistent with the result of Olander and Vitousek (2000)^23^, who proposed that there was negative feedback mechanism between P availability and AcP, namely, AcP activity was suppressed by high P availability. Differently, both AP content and AcP activity were all lower in NR3 than in ER under 0.25–1 mm and 1–2 mm. The relative low amounts of soil microbe and roots in relatively smaller aggregate fraction may result in lower AcP activity and then lower AP release. Therefore, it can be concluded that AcP had an important regulation effect on soil available P in different aggregate fraction scale.

There was no evident response of nematodes to row spacing changes. In above-mentioned results, row spacings did not alter soil organic carbon which is a key driving factor for soil biota^24,25^. The whole nematode community were all significantly and positively related to SOC content in our study (r = 0.35, P < 0.05, Fig. S4). Therefore, there was the similar response to row spacing between soil nematodes and soil carbon. Additionally, the nematodes respond distinctly to aggregate fractions, with the richness and abundance of nematode community being decreased in 0.25–1 mm and <0.25 mm. The size of aggregates is one of important factors that regulate nematode distribution, and the nematode with relatively large size would be limited by small-size aggregate. For example, only one genera of BF was found in <0.25 mm aggregates. Soil aggregates with different sizes offer spatially heterogeneous habitats for soil nematodes^26^. As presented by hierarchical clustering analysis, the more nematode guilds with cp value 4–5 belonged to *K* strategists indicated a relatively stable environment under NR3 within 0.25–1 mm aggregate. This result was further confirmed by nematode faunal analysis, which also suggested that there was less perturbation in NR3 within 0.25–1 mm. These results proved that NR3 had potential to construct more stable and structural food web, and therefore possibly provided better ecosystem services and directly contribute to grain yield.

Most researches on the relationship between multifunctionality and biodiversity have been carried out^27–29^, and a few research was focused on it in aggregate scale. In our study, aggregate fractions were significantly and positively related to multifunctionality. Multifunctionality were significantly lower in <0.25 mm than in 1–2 mm and 0.25–1 mm, which may relate to the soil properties at different aggregate levels. According to Peng *et al*.^20^ and Zheng *et al*.^30^, SOC as the primary food and energy sources of soil organisms contributed more to that in the larger aggregates (>0.25 mm). The low values of SOC in <0.25 mm may weaken the activity of soil organisms and then multifunctionality. Furthermore, soil aggregates as the essential features of soil structure are strongly shaped by the spatial distribution of soil biota. According to structural equation model (SEM), soil aggregate fractions indirectly affected multifunctionality via controlling the richness of trophic group and the whole nematode community. The relative nutrient-poor conditions and the limited living spaces in <0.25 mm aggregate would prevent most organisms with large-body and higher trophic level from entering the relative smaller aggregate, therefore reduced the biotic interactions among soil food web and then ecosystem functioning.

Row spacing patterns directly impacted multifunctionality as suggested by structural equation model (SEM). Among the indicators of multifunctionality, Random Forest analysis indicated total phosphorus (TP), available phosphorus (AP) and β-N-acetylglucosaminidase (NAG) were more important than other indicators. The row spacing effect on TP and AP was significant, but not on NAG. Therefore, it was concluded that row spacing had effect on multifunctionality mainly through modulating TP and AP. For different row spacing patterns, NR3 had higher value of multifunctionality, which was also attributed to the higher amount of TP and lower one of AP in 1–2 mm and 0.25–1 mm. Higher TP and lower AP values are considered higher functioning (Table S1).

## Conclusions

In conclusion, row spacing did not impact soil C, N and pH, but influence P and acid phosphatase (AcP), with AcP activity and TP being lower and AP higher in >2 mm aggregate fraction under NR3. Soil nematodes were more sensitive to aggregate fraction than row spacing, with both richness and abundance of nematode community, Ba, PP, and the richness of Fu being decreased in <1 mm aggregate fraction. We found that higher total phosphorus and lower available phosphorus are the main contributed indicators of multifunctionality in NR3 which resulted in higher multifunctionality in 1–2 mm and 0.25–1 mm. Nematode faunal analysis and hierarchical clustering analysis showed that 0.25–1 mm in NR3 was less disturbed or relatively undisturbed environments. Structural equation model suggested that row spacing pattern had directly positive effect on multifunctionality, while aggregate fractions indirectly impacted multifunctionality mainly by controlling the richness of trophic group and total nematode community. Compared with other row spacings, NR3 (with 60 cm + 40 cm non-equidistant-row) was a suitable planting pattern because of its potential to construct more stable and structured food web.

## Materials and methods

### Study site and soil sampling

**Figure 5:**
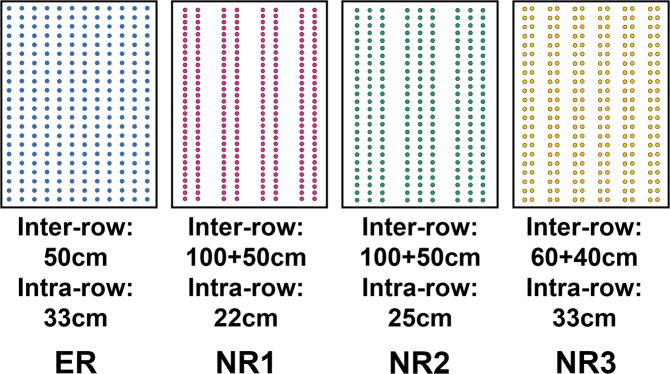
Schematic diagrams of four row spacing patterns.

This study was carried out at the Fuxin Agricultural Research Station of Liaoning Academy of Agricultural Sciences, Northeast China (42°11′N, 121°70'E). The mean annual temperature and precipitation are 7.2 °C and 705 mm, respectively. The row spacing pattern experiment was initiated in 2014. The soil is classified as cinnamon soil (Luvisols in FAO system). The experiment followed a complete randomized design with four treatments for different row spacing patterns including equidistant-row spacing as the control (ER)(12 × 24 plants/plot, equal inter-row distance with 50 cm, intra-row distance between plants in each row with about 33 cm), (ER), and three types of non-equidistant-row spacing NR1(8 × 36 plants/plot, non-equidistant-row distance with 100 cm + 50 cm, intra-row with about 22 cm), NR2 (9 × 32 plants/plot, non-equidistant-row distance with 100 cm + 50 cm, intra-row with 25 cm) and NR3 (12 × 24 plants/plot, non-equidistant-row distance with 60 cm + 40 cm, intra-row with about 33 cm) (Fig. 5). The experiment was a completely randomized block design with three replicates for each treatment. Total 12 plots were set and each plot with 48 m^2^ (6 m × 8 m) had the same plant population density with about 60,000 plants ha^−1^ (288 plants/plot) in all treatments. Maize (*Zea mays* L.) was sown in early May and harvested in late September followed by conventional tillage and no pesticides were used during the study period. Total 150 kg ha^−1^ (NH_4_)_2_HPO_4_ (N-P_2_O_5_, 18–46%) and 150 kg ha^−1^ compound fertilizer (N-P_2_O_5_-K_2_O, 26–12–12%) were applied at sowing stage and 450 kg ha^−1^ urea at jointing stage.

Soil samples were randomly collected from the ploughed layer (0–20 cm) of each plot on October 27, 2017 after the harvest. In each plot, two random undisturbed soil blocks (each 20 cm length, 15 cm width and 15 cm depth) on the row were collected for the analysis of soil aggregation. Two soil blocks in each plot were homogenized. Fresh samples were brought to laboratory and stored at 4 °C until processing and analyses.

Soil aggregate fractions were separated using the sieving method^31^. The field-moist soil was dried at 4 °C until it reached a gravimetric water of about 100 g H_2_O kg^−1^^32^. Then the soils were sieved (5 mm mesh) to remove plant material and roots. Then the aggregates were separated by placing about 100 g of soil (<5 mm) each time on top of a sieve nest mounted on a Retsch AS200 Control (Retsch Technology, Düsseldorf, Germany), repeating until all the collected soil was sieved. After mechanically shaking the sieves for two minutes at 1.5 mm amplitude, the soils were separated into the following aggregate fractions, i.e. >2 mm (large macroaggregates), 1–2 mm (macroaggregates), 0.25–1 mm (small macroaggregates) and <0.25 mm (microaggregates, and silt and clay fractions)^33,34^.

### Extraction and identification of soil nematodes

Nematodes were extracted from 50 g fresh soil collected from each aggregate fraction of each replicate using a modified cotton-wool filter method^35^. All nematodes in each sample were counted and at least 100 specimens per sample were identified to genus level using a microscope (OLYMPUS BX51) according to Bongers^36^. Then nematodes were classified into four groups (bacterivores (Ba), fungivores (Fu), plant- parasites (PP) and omnivore-predators (OP)) and different functional guilds with cp (colonizer-persister) value 1–5, richness, and Enrichment index (EI) and structure index (SI) were calculated according to Ferris *et al*.^37^ and http://nemaplex.ucdavis.edu/Ecology/EcophysiologyParms/GenusParmsQuery.aspx. The richness of nematode communities is the total number of taxa in each sample, and that of the four groups are calculated according to their respective taxa number.

### Measurement of individual ecosystem functions

Eight indicators were measured to indicate ecosystem functions, including β-N-acetylglucosaminidase (NAG, indicating the degradation of chitin^13^), acid phosphatase (AcP, indicator of hydrolyzing phosphomonoesters to release phosphate^13^), and soil organic carbon (SOC), total nitrogen (TN), alkaline nitrogen (AN), total phosphorus (TP) and available phosphorus (A-P) related to soil nutrient stock and turnover. The two soil extracellular enzyme activities (NAG and AcP) were measured from 2 g of fresh soil by fluorometry^38,39^. SOC and TN were measured from 0.5 g of air-dried soil by an automatic elemental analyzer (Elemental Analyzer System Vario MACRO cube, Germany). Alkaline nitrogen (AN) was measured from 2 g of air-dried soil as described in Bremner *et al*.^40^. was TP was measured from 1 g of air-dried soil by persulfate oxidation followed by colorimetric analysis^41^. AP was extracted from 1 g of air-dried soil by 0.5 M NaHCO_3_^42^. Soil pH was determined from 5 g of air-dried soil with a glass electrode in 1: 2.5 soil: water (w/v).

### Assessing multifunctionality

We used multivariate measure approach to calculate a multifunctionality as described in Meyer *et al*.^43^. Random Forest analysis was conducted to identify the main contributors of multifunctionality among the ecosystem indicators (NAG, AcP, SOC, TN, AN, TP and AP). The orientation based on the biological meaning of each ecosystem indicators were show in Table S1.

### Statistical analysis

Two-way analysis of variance was used to test the main and interactive effects of row spacing patterns and aggregate fractions on nematode community composition (the richness and abundance of nematode community and all trophic groups), ecosystem function indicators (NAG, AcP, SOC, TN, AN, TP and AP) and multifunctionality. If necessary, nematode data were ln (x + 1) transformed to meet normality prior to statistical analysis. LSD multiple comparison tests were used when main effects and/or interactive effects were significant. Differences at P < 0.05 level were considered significant. SPSS version 19.0 (SPSS Inc., Chicago, IL) was performed for these analyses. Hierarchical clustering analysis were used to evaluate the differences of nematode community composition within aggregate fractions by using R package *pheatmap*^44^ in R (version 3.6.2, R Core Development Team, 2019). Random Forest analyses were conducted using the R package *randomForest*^45^, P-value and the cross-validated R^2^ were assessed with 5,000 permutations of the indicators of multifunctionality using the R package *A3*^46^. Structural equation model (SEM) was used to identify the direct and indirect effect of row spacing patterns and soil aggregate fractions on the relationship between soil nematode community and multifunctionality by using the Amos 17.0 software^47^. Spearman correlations was conducted between aggregate fractions, soil pH, ecosystem indicators, richness and abundance of total nematode and trophic groups, and multifunctionality by using R package *PerformanceAnalytics*^48^.

## Supplementary information

Supplementary information
