# Supplementary material for: Effect of row spacings on soil nematode communities and ecosystem multifunctionality at an aggregate scale
Source: Sci Rep. 2020 Mar 16;10:4779. doi: 10.1038/s41598-020-61498-x (PMC7076006; doi:10.1038/s41598-020-61498-x)
Supplement: Supplementary file 1 — Supplementary information [file 41598_2020_61498_MOESM1_ESM.docx]

**Supplementary information for:**

**Effect of row spacings on soil nematode communities and ecosystem multifunctionality at an aggregate scale**

**Guizong Zhang^1, 4^, Xinchang Kou^1, 3^****, Xiaoke Zhang^1^*****, Wei Bai^2^, Wenju Liang^1^***

^1^ Institute of Applied Ecology, Chinese Academy of Sciences, Shenyang 110016, China

^2^ Tillage and Cultivation Research Institute, Liaoning Academy of Agricultural Sciences, Shenyang 110161, China

^3^ School of Geographical Science, Northeast Normal University, Changchun 130024, China

^4^ University of Chinese Academy of Sciences, Beijing 100049, China

***Corresponding author. E-mail:** [**liangwj@iae.ac.cn**](mailto:liangwj@iae.ac.cn)**;** [**zxk@iae.ac.cn**](mailto:zxk@iae.ac.cn)

**Suppl. Table S1: List of ecosystem variables measured in the experimental plot included in the analysis. Direction of better: Variable indicating that higher values are considered higher functioning (1), lower values are considered higher functioning (-1) or no clear direction can be defined (0).**

| Abbreviation | Variable | Approximated ecosystem function | Direction of better |
| --- | --- | --- | --- |
| AN | alkaline nitrogen | Nitrogen cycling, nutrients left after maize growth, potential for nitrogen  leaching | -1 |
|  |  |  |  |
| NAG | β-N-acetylglucosaminidase | Nitrogen cycling, indication of chitin degradation | 1 |
|  |  |  |  |
| AcP | acid phosphatase | Phosphorus cycling, indication of phosphorus mineralization | 1 |
|  |  |  |  |
| SOC | soil organic carbon | Soil fertility, carbon sequestration | 1 |
|  |  |  |  |
| TN | total nitrogen | Soil fertility | 1 |
|  |  |  |  |
| TP | total phosphorus | Soil fertility | 1 |
|  |  |  |  |
| AP | available phosphorus | Phosphorus cycling, nutrients left after maize growth, potential for phosphorus leaching | -1 |


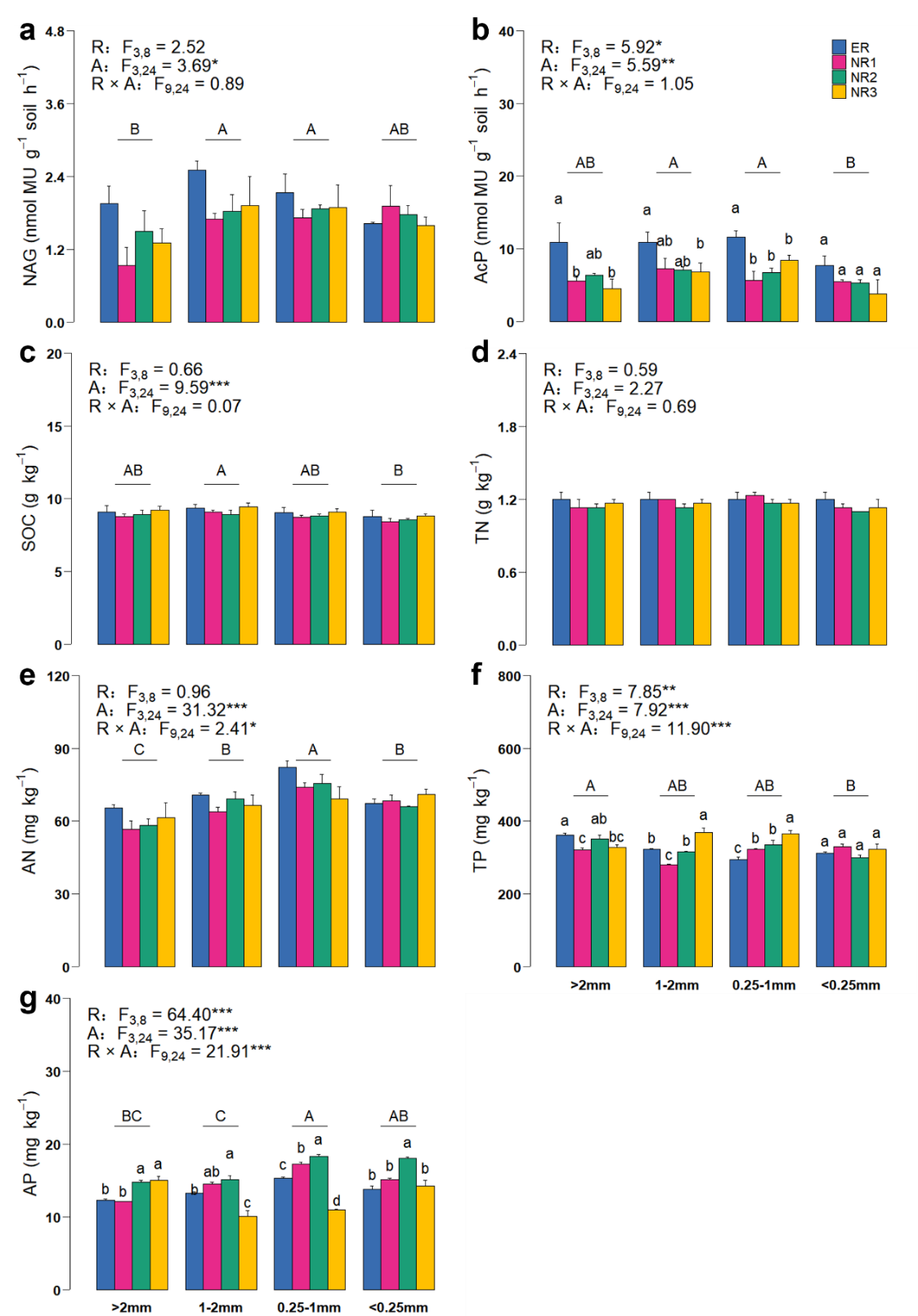


**Fig S1:** Effects of row spacing on eight indicators of ecosystem functions: β-N-acetylglucosaminidase (NAG, **a**), acid phosphatase (AcP, **b**), soil organic carbon (SOC, **c**), total nitrogen (TN, **d**), alkaline nitrogen (AN, **e**), total phosphorus (TP, **f**), available phosphorus (AP, **g**) and. Bars indicate standard errors. F and P values from a two-way ANOVA on the effects of row spacing (R) and aggregate fraction (A) and their interactions (R×A) are also presented. Significance levels are as follows: *P < 0.05, **P < 0.01, ***P < 0.001. Different uppercase letters on the horizontal line indicate significant differences among aggregate fractions at P < 0.05; different lowercase letters above bars indicate significant differences among row spacings in the same aggregate fractions at P < 0.05.

**
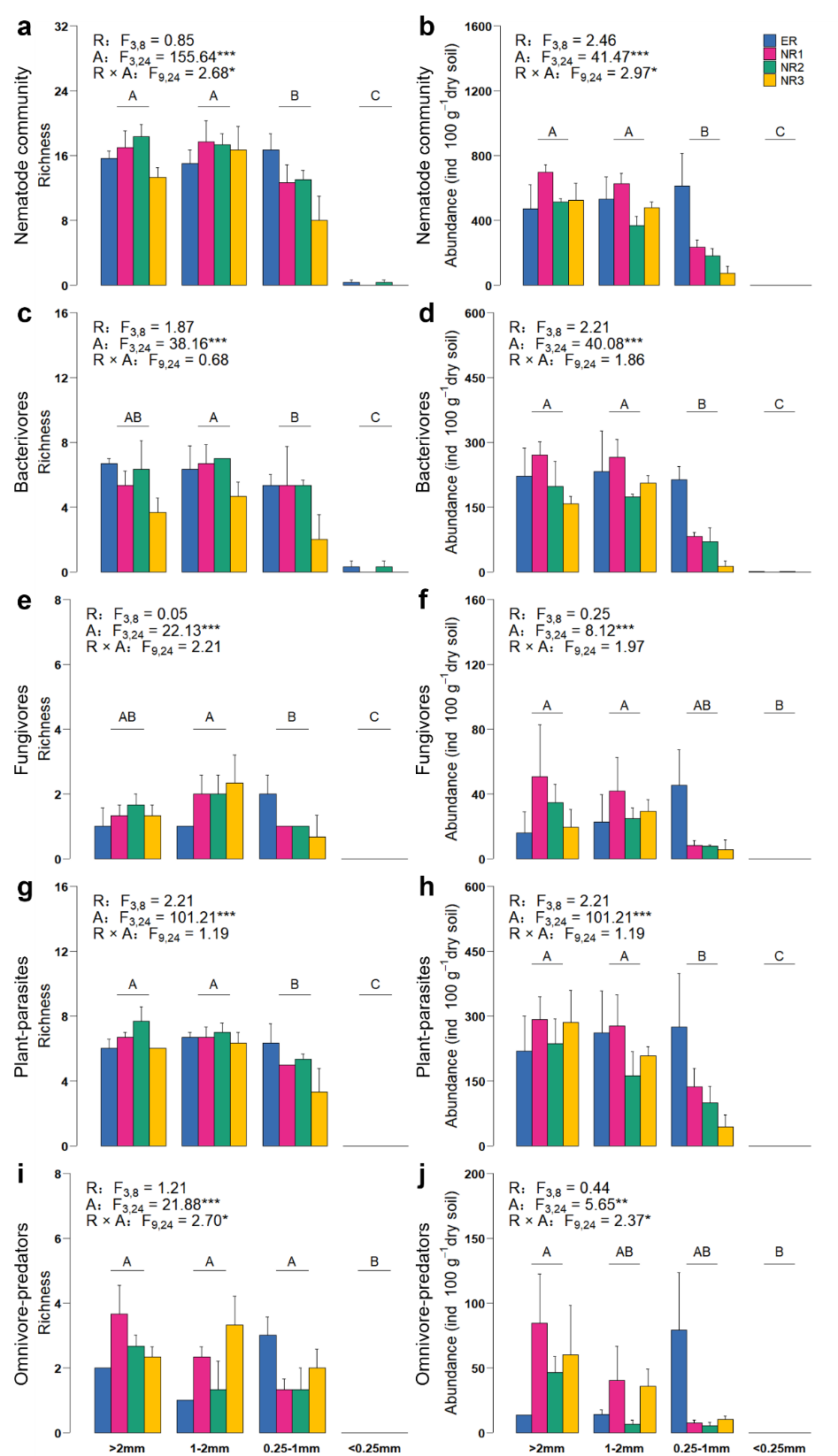
**

**Fig S2:** Effects of row spacing on richness and abundance of nematode community (**a**, **b**), bacterivores (**c**, **d**), fungivores (**e**, **f**), plant-parasites (**g**, **h**), omnivore-predators (**i**, **j**) within aggregates. Bars indicate standard errors. F and P values from a two-way ANOVA on the effects of row spacing (R) and aggregate fraction (A) and their interactions (R×A) are also presented. Significance levels are as follows: *P < 0.05, **P < 0.01, ***P < 0.001. Different uppercase letters on the horizontal line indicate significant differences among aggregate fractions regardless of row spacing at P < 0.05.


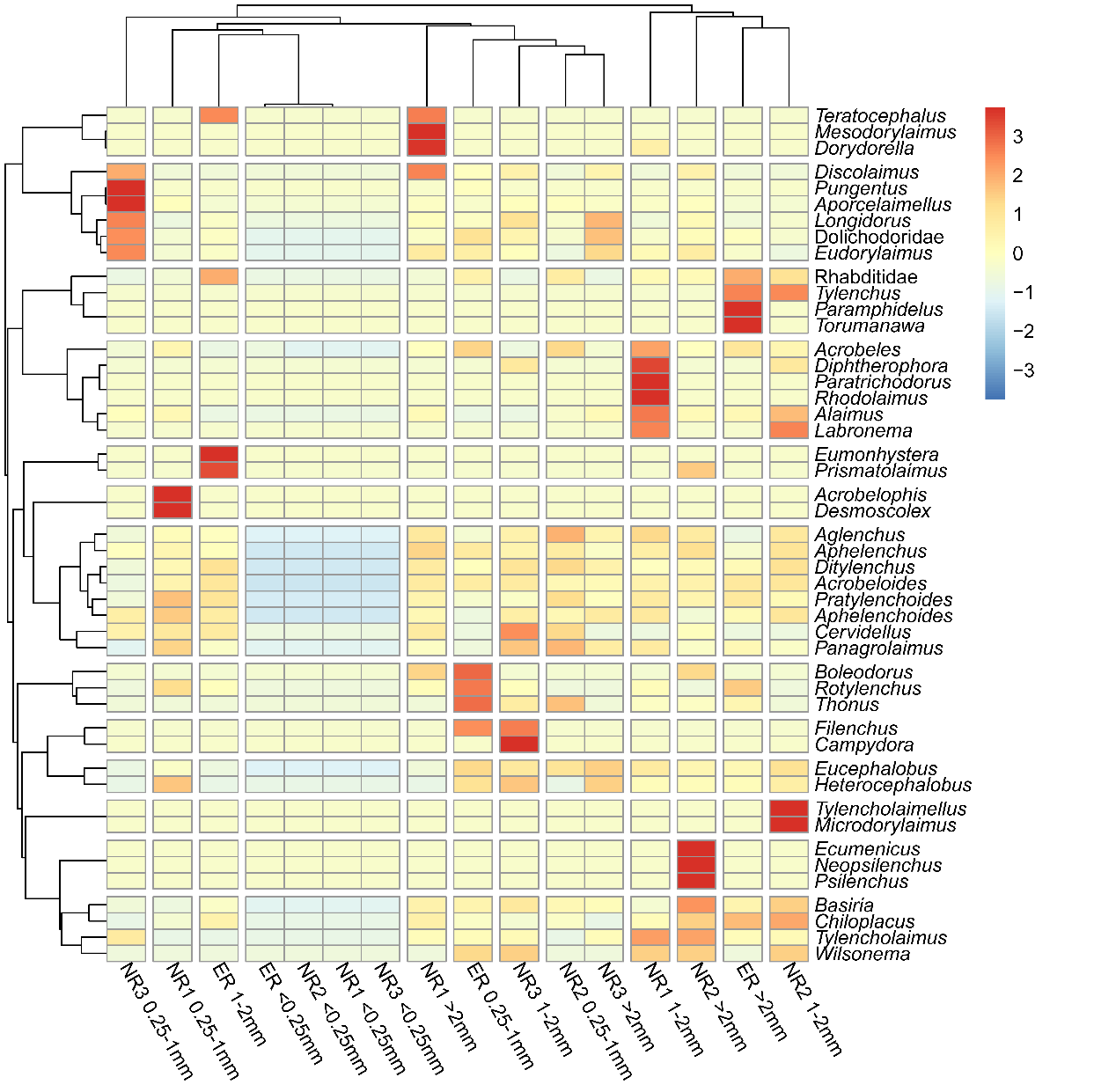
 **Fig S3:** The result of hierarchical clustering analysis for different aggregate sizes under the four row spacing treatments. Red and blue indicate higher abundance and lower abundance, respectively.


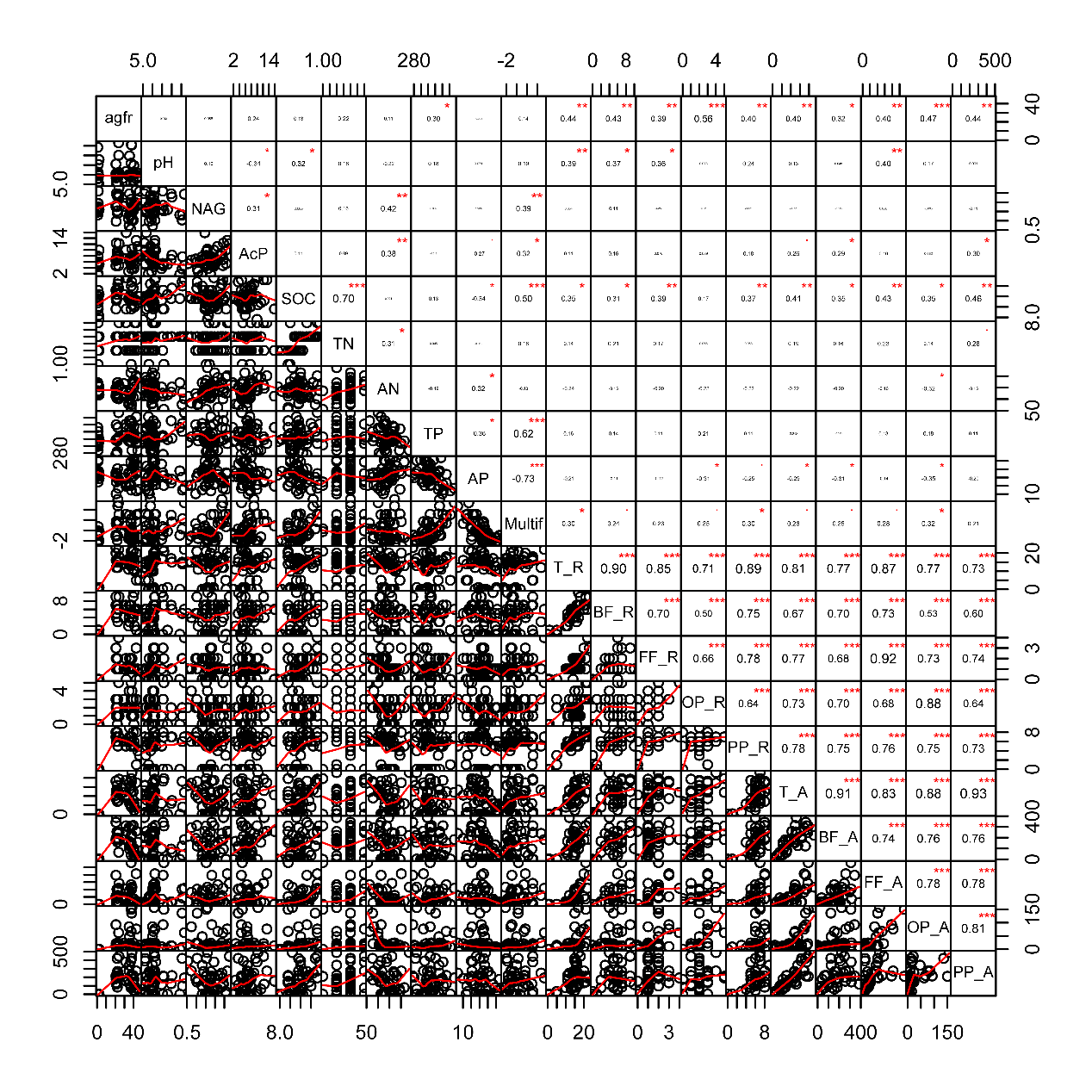


**Fig S4:** Spearman correlations between aggregate fractions (agfr), soil pH, ecosystem indicators (NAG, AcP, SOC, TN, AN, TP, AP), richness (R) /abundance (A) of total nematode and trophic groups, and multifunctionality (Multif). BF: bacterivores; FF: fungivores; OP: omnivore-predators; PP: plant-parasites.
